# Light exposure during development affects physiology of adults in *Drosophila melanogaster*

**DOI:** 10.3389/fphys.2022.1008154

**Published:** 2022-11-25

**Authors:** Milena Damulewicz, Aleksandra Tyszka, Elzbieta Pyza

**Affiliations:** Department of Cell Biology and Imaging, Institute of Zoology and Biomedical Research, Faculty of Biology, Jagiellonian University, Krakow, Poland

**Keywords:** clock, sleep, dopamine pathway, histamine, octopamine, cryptochrome, mushroom bodies

## Abstract

Light is one of most important factors synchronizing organisms to day/night cycles in the environment. In *Drosophila* it is received through compound eyes, Hofbauer-Buchner eyelet, ocelli, using phospholipase C-dependent phototransduction and by deep brain photoreceptors, like Cryptochrome. Even a single light pulse during early life induces larval-time memory, which synchronizes the circadian clock and maintains daily rhythms in adult flies. In this study we investigated several processes in adult flies after maintaining their embryos, larvae and pupae in constant darkness (DD) until eclosion. We found that the lack of external light during development affects sleep time, by reduction of night sleep, and in effect shift to the daytime. However, disruption of internal CRY- dependent photoreception annuls this effect. We also observed changes in the expression of genes encoding neurotransmitters and their receptors between flies kept in different light regime. In addition, the lack of light during development results in decreasing size of mushroom bodies, involved in sleep regulation. Taking together, our results show that presence of light during early life plays a key role in brain development and affects adult behavior.

## Introduction

Light plays a key role in the synchronization of animal behavior to cyclical changes in environmental conditions, it acts as an arousal signal for diurnal animals and promotes sleep in nocturnal ones (Redlin, 2001). *Drosophila* is a very useful model for sleep/activity research since it exhibits a clear daily activity pattern. Under LD12:12 (12 h of light and 12 h of darkness) conditions it shows two peaks of activity, morning and evening, while in the middle of the day and night it has resting time called siesta and sleep, respectively. This rhythmic behavior is regulated by brain sleep centers, glial cells, as well as by different clusters of clock cells (Grima et al., 2004; Stoleru et al., 2004; Picot et al., 2007; Cusumano et al., 2009; Zhang et al., 2009; Yao and Shafer, 2014; Chatterjee et al., 2018; Guo et al., 2018; Schlichting et al., 2019). Sleep in flies is defined as at least 5 min of total inactivity with elevated sensory threshold and specific body posture (Shaw, 2000). Daytime (siesta) and nighttime sleep, which are the deepest around noon, and at midnight, respectively constitute total sleep. Siesta is usually less deep and has shorter single sleep episodes as well as lower arousal threshold comparing to nighttime sleep (Hendricks et al., 2000; Huber et al., 2004).

Sleep is regulated by several neurotransmitters: promoting sleep as serotonin and gamma-aminobutyric acid (GABA) (Yuan et al., 2005, 2006; Chung et al., 2009; Haynes et al., 2015; Qian et al., 2017) and wake-promoting as dopamine, octopamine, and histamine (Friggi-Grelin et al., 2003; Hamasaka and Nässel, 2006; Pantazis et al., 2008; Mao and Davis, 2009; Crocker et al., 2010; Liu et al., 2012; Ueno et al., 2012; Oh et al., 2013; Sitaraman et al., 2015; Alejevski et al., 2019) and those playing a dual role depending on target cells (acetylcholine, glutamate) (Buchner et al., 1986; Yasuyama and Salvaterra, 1999; Sinakevitch-Pean et al., 2001; McCarthy et al., 2011; Raghu and Borst, 2011; Takemura et al., 2011; Liu and Wilson, 2013; Mauss et al., 2014, 2015; Tomita et al., 2015; Robinson et al., 2016). In addition, there are neurotransmitters regulating clock network activity: Pigment-dispersing factor (PDF) and short Neuropeptide F (sNFP), which play a role in the integration of clock and sleep (Shang et al., 2013; Kunst et al., 2014).

Dopamine is a monoamine synthesized from tyrosine or phenylalanine mainly in a set of specific neurons in the presence of tyrosine hydroxylase encoded in *Drosophila* by the *ple* gene. Once produced and released into synaptic cleft it can bind to specific receptors on postsynaptic cells in the following structures involved in sleep regulation: Dop1R1 in neurons of mushroom bodies, ellipsoid body and fan-shaped body, Dop1R2 in neurons of mushroom bodies and DD2R in three pairs of dorsal neurons in the ventral nerve cord and sleep promoting protocerebral bridge interneurons (Friggi-Grelin et al., 2003; Mao and Davis, 2009; Liu et al., 2012; Ueno et al., 2012; Sitaraman et al., 2015; Tomita et al., 2021).

The another neurotransmitter involved in light transmission and sleep regulation is histamine. It is released mostly by the retina photoreceptors and synthesized by histidine decarboxylase (Hdc). One of its receptor HisCl1 is expressed in photoreceptors and clock neurons, and the other one—Ort receptor—in the lamina and medulla interneurons (Hamasaka and Nässel, 2006; Pantazis et al., 2008; Oh et al., 2013; Alejevski et al., 2019).

Octopamine is synthesized by anterior paired lateral neurons (APL) using tyramine beta hydroxylase (Tbh) and its signal is transmitted to mushroom bodies through Oamb receptor (Crocker et al., 2010).

All these neurotransmitters control activity of specific neurons located in structures called sleep centers. The most important ones are mushroom bodies (MB) and central complex in the central and dorsal brain (Joiner et al., 2006; Pitman et al., 2006; Donlea et al., 2011; Liu et al., 2012; Guo et al., 2016). The mushroom body is formed by Kenyon cells (Kc), and can be divided into morphologically distinct parts: calyx, which contains synapses between Kc and projection neurons, and five output lobes (α, α′, β, β’, γ), composed of synapses between Kc, mushroom body output neurons and dopaminergic neurons (Aso et al., 2014). Output signals from multiple sensory pathways are received by calyx and then transferred to the lobes. MB integrate and process also visual signals coming from medulla, lobula and posterior lateral protocerebrum (Morante and Desplan, 2008; Vogt et al., 2016; Li et al., 2020).

Light input is received by the visual system: retinal photoreceptors, Hofbauer-Buchner eyelet and ocelli through the rhodopsin-dependent phototransduction pathway (Busto et al., 1999; Hassan et al., 2000). All photoreceptors use histamine to communicate with downstream neurons (Hamasaka and Nässel, 2006), but some of them use also acetylcholine as a neurotransmitter, for example eyelets which contact directly with clock cells (Pollack and Hofbauer, 1991; Yasuyama and Meinertzhagen, 1999; Helfrich-Förster et al., 2002; Malpel et al., 2002; Damulewicz et al., 2020). In addition, light is detected by deep brain photoreceptors, Cryptochrome (CRY) and Quasimodo (QSM), which are mostly involved in clock synchronization (VanVickle-Chavez and Van Gelder, 2007; Chen et al., 2011). Moreover, rhodopsin 7, which is involved in non-visual light signaling, regulates morning activity and time of siesta (Kistenpfennig et al., 2017).

In larvae light activates CRY located in clock neurons (four LNvs and DN1s) and photoreceptors of the Bolwig organ (BO), which contains rhodopsin 5 or rhodopsin 6 (Malpel et al., 2002; Mazzoni et al., 2005) and communicates directly with the larval pacemaker to synchronize this oscillator to changes in environmental light conditions (Keene et al., 2011; Klarsfeld et al., 2011). Although larvae do not show circadian behavior, their clock is entrained by light which can affect the clock in adult flies. It has been shown that a single light pulse during embryonal or postembryonic development can synchronize activity of adult flies kept in constant darkness (Sehgal et al., 1992; Zhao et al., 2019).

The role of light in the sleep regulation is already well described, however, little is known about the link between light exposure during development and sleep amount in adult life. In this study, using *Drosophila* as a model, we showed that lack of light in early life affects behavior of adult flies.

## Material and methods

### Flies

The following strains of *Drosophila melanogaster* were used: wild type CantonS, *norpA* ^*7*^ mutant (BDSC, no. 5685), *cry* ^*01*^ mutant (Dolezelova et al., 2007), OK107-Gal4 (kindly donated by P. Menegazzi, Wurzburg University), UAS-mCD8::GFP (BDSC, stock no. 5137).

Flies were maintained on a standard cornmeal medium at constant temperature 25°C. The control group was kept under LD12:12 (12 h of light and 12 h of darkness) regime during development and adulthood (group described as LD/LD), while experimental flies were kept in constant darkness (DD) during development (embryonal, larval and pupal stage) (group described here as DD/DD). Because sleep assay was measured on the second day of LD12:12 conditions, we added the additional group called DD/LD, in which flies exposed to DD during development were transferred for 2 days to LD12:12.

### Behavioral assays

Locomotor activity was recorded using *Drosophila* Activity Monitoring System (DAMS, Trikinetics, Waltham) for 5 days in LD12:12 and 6 days in DD. Activity was counted every 1 min and analyzed in Excel using “Befly!” software (Department of Genetics, Leicester University). Lomb—Scargle normalized periodogram was used to determine rhythmic flies; individuals with period below 10 (confidence level 0.05) were regarded as arrhythmic. Flies, which did not survive until the end of experiments were removed from analyses. Every experiment was repeated three times, at least 60 flies in total were used.

Sleep analysis was performed on the second day of LD12:12, and sleep was recorded as at least 5 min of a fly immobility.

### Immunohistochemistry

Males from the strain OK107 > GFP were collected at ZT1 (1 hour after lights-on in LD 12:12) or CT1 (in DD), their heads were fixed in 4% paraformaldehyde and brains were isolated. GFP fluorescence of this strain is strong enough to skip additional staining steps.

For TH staining wild type flies were used. Fixed brains were washed in PBS and 0.2% PBST (PBS with Triton X), then unspecific antigens were blocked with Normal Goat Serum (NGS) for 1 h. Samples were incubated with mouse anti-TH antibodies (1:1000, Immunostar) for 3 days at 4°C. Then, brains were washed three times in 0.2% PBST and incubated for 2 h with goat anti-mouse antibodies conjugated with Cy3 (1:500, ThermoFisher). After washing in 0.2% PBST, brains were mounted in Vectashield medium (Vector) and examined with a Zeiss Meta 510 Laser Scanning Microscope.

The size of the brain and GFP-labelled mushroom bodies lobes was measured using ImageJ software (Area measurements). To avoid changes in body size, dependent on overcrowding, the same number of parental flies was used for every group.

The TH immunofluorescence intensity was measured for specific dopaminergic cells: PPL1, PAM and PPM3 because these cluster are known to innervate sleep centers. Area of every single cell was selected manually and mean grey value was measured using ImageJ software.

Experiments were repeated 3 times and each repetition was composed of 10 brains per group.

### Gene expression analysis by qPCR

For gene expression analysis we divided the experimental flies into two groups—first was continuously kept in constant darkness (DD/DD), the second one was moved to LD12:12 for 2 days (DD/LD) to provide light input and synchronize flies. All groups were collected at the same time—ZT1 (1 hour after lights-on for control and flies transferred to LD12:12) and CT1 (for flies kept in DD). At least 20 heads were cut off and the total RNA was isolated using a TriReagent (Invitrogen). 1 μg of the total RNA was used to prepare cDNA with the High Capacity cDNA Reverse Transcription Kit (Thermo Fisher Scientific) and random primers. cDNA (diluted 1:10) was used for SybrGreen qPCR (KapaBiosystems) analysis. The specific primers (the specificity was controlled with Primer BLAST and gel electrophoresis) used for the reaction are listed in Table 1. Expression level was calculated by relative standard curve. The reference gene used was *rpl32*. Experiments were repeated 5 times.

**TABLE 1:** Primer sequences used for qPCR.

Gene	Primer sequence	Product length [bp]
*Dop1R1*	For: GGA​TAC​AAT​AGT​TGG​CAT​CTT​CCT	123
	Rev: ACG​AGG​CCA​GGA​ACA​GAT​TG	
*Dop1R2*	For: GGT​GTC​GAT​AAC​TCC​AGC​GT	101
	Rev: GTC​GAT​AGC​GTC​GAG​TCG​TT	
*DD2R*	For: TGT​CGA​TAA​CTC​CAG​CGT​CG	98
	Rev: TCG​ATA​GCG​TCG​AGT​CGT​TG	
*ple*	For: ATT​GCA​TTG​TGC​AGG​CTC​AG	96
	Rev: ATT​CCT​CCA​AGG​GCA​GGT​TC	
*HisCl1*	For: GCG​CCA​TGA​AGT​TTG​AGT​CC	106
	Rev: ACG​GGA​TTT​GCA​GGG​TGT​AG	
*Ort*	For: CCA​CAC​CAC​GGA​TGA​CTT​GA	99
	Rev: TCA​TTT​CGG​ATG​AGG​GCC​AC	
*Oamb*	For: TTC​CAA​GGG​CAT​CGG​TTC​TC	80
	Rev: CCT​GCT​GGT​TTG​ATC​CTC​GT	
*Hdc*	For: TCA​AGC​GTG​CAT​TTC​ATC​AG	136
	Rev: TAC​ACA​GAT​ACT​TGC​CGA​GC	
*Tbh*	For: CGA​TGG​ACT​TCG​CCT​ATC​A	137
	Rev: CAG​ATC​GGT​GGC​GGA​ATT​AT	
*Pdf*	For: GGC​CAC​TCT​CTG​TCG​CTA​TC	84
	Rev: ATT​AGC​TCC​GAG​TTG​CGC​TT	
*Pdfr*	For: ACG​GCC​GTG​TTA​CAA​GCC​G	124
	Rev: GGA​GAG​GCA​GAG​GCC​CAC​GA	
*sNPF*	For: ACC​GCC​TAT​GAT​GAC​CTC​CT	115
	Rev: CGT​ACA​GTC​CGT​CGT​AGT​CG	
*sNPF-R*	For: TGC​CAT​CGA​TCG​GTA​CTT​CG	98
	Rev: GGG​CTA​TCA​CCC​AGA​TGC​TC	
*rpl32*	For: ATG​CTA​AGC​TGT​CGC​ACA​AAT​G	107
	Rev: GTT​CGA​TCC​GTA​ACC​GAT​GT	

### Statistical analysis

GraphPad Prism software was used for statistics and making graphs. Outliers were removed using Grubbs’ test (GraphPad online software). Shapiro-Wilk’s test was used to check normality in distribution. Data were analyzed using one-way ANOVA with post-hoc Tukey’s test or *t*-test to detect statistically significant differences between groups.

## Results

**FIGURE 1:**
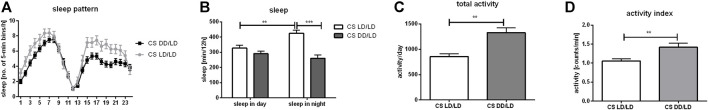
Light during embryonal/larval stage regulates sleep. Wild-type flies (Canton S, CS) were grown starting from embryo in LD12:12 or DD regime. After eclosion all flies were kept in LD12:12 and 2-days old males were used for DAMS locomotor analysis. Sleep and activity level were measured during second day of experiment. **(A)** Sleep profile of flies (measured as number of 5-min beans per hour) maintained without light during early life (DD/LD) shows differences with control (LD/LD) during first 8 h after light-on and during whole night. **(B)** Sleep time measured as number of minutes per 12 h showed that DD/LD group has decreased level of sleep during the night. Control flies show higher level of sleep during the night than during siesta, while experimental insects have similar level of sleep throughout the day. **(C)** Total activity measured as number of minutes per 24 h was increased in flies which spend early life in darkness compare with control. **(D)** Activity index measured as ratio of total activity per day and number of minutes awaked. Mean ± SEM. Statistically significant differences shown as asterisks **p ≤ 0.01, ***p ≤ 0.001.

Light exposure during development is not necessary to maintain rhythmicity in behavior, but it affects activity/sleep ratio. It has been shown that larvae kept in DD can be easily synchronized to light:dark regime, which suggests that their clock develops normally. Our data confirmed these results because flies which were kept in DD during development and transferred to LD12:12 after eclosion were perfectly synchronized and after moving back to DD they were able to maintain 24 h rhythmicity. We focused our research on the other aspect of behavior ‒ activity and sleep amount and pattern. We observed that flies from the experimental group (DD/LD) were more active than the control (LD/LD) (*p* = 0.0052), activity index was increased (*p* = 0.0025) and their sleep amount during the night, measured in minutes, was decreased (*p* = 0.0202), while siesta was not changed (*p* = 0.0723) (Figure 1). Moreover, control flies had longer sleep during the night than during the day, while experimental ones had similar sleep level all over the day.

### Neurotransmission is affected by the lack of light during development

Sleep is regulated by several neurotransmitters released by specific neurons and recognized by receptors on target cells. Because we observed increased activity and decreased sleep level in the experimental flies, we expected to see changes in the expression of wake-promoting factors. The most important ones are dopamine, histamine and octopamine, as well as PDF and sNPF which affect the clock network. To investigate how light during development regulates these pathways we decided to check changes in the mRNA level of both, neurotransmitters and their receptors in heads. We compared mRNA level of selected genes in adult flies from three different groups: flies which were maintained during the development and as adults in DD (group DD/DD), flies developing in DD and transferred to LD12:12 as adults, which is similar to the experiment for sleep analysis (group DD/LD) and control flies developing and kept in LD12:12 after eclosion (group LD/LD).

**FIGURE 2:**
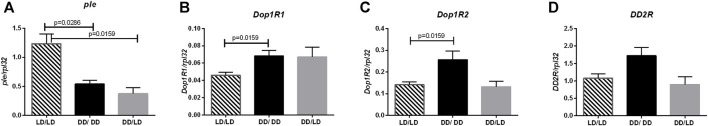
Darkness affects dopamine pathway. qPCR analysis of expression level of genes connected with dopamine signaling for flies maintained in normal conditions LD12:12 (LD/LD), maintained in DD (DD/DD) and developed in darkness and after eclosion transferred to LD12:12 (DD/LD). Expression checked for: **(A)** *tyrosine hydroxylase* (*ple*), **(B)** dopamine receptor *Dop1R1*, **(C)** *Dop1R2* and **(D)** *DD2R*. *N* = 5. Mean ± SEM.

First, we investigated effect of light during development on dopamine signaling. Our results showed that dopamine synthesis was decreased in flies developing in constant darkness (Figure 2A, LD/LD vs. DD/LD *p* = 0.00159, LD/LD vs. DD/DD *p* = 0.0286), which suggests that light during development is necessary to maintain high level of dopamine signaling. This result was not expected, as dopamine is wake-promoting factor, and knowing that experimental flies are more active we predicted to observe higher dopamine synthesis. However, the opposite situation was observed for *Dop1R1*, which was expressed at higher level in the groups DD/LD and DD/DD (Figure 2B, LD/LD vs. DD/LD *p* = 0.1905, LD/LD vs. DD/DD *p* = 0.0159). The level of *Dop1R2* mRNA was increased in the group DD/DD, but it was decreased after moving flies to LD12:12 (Figure 2C, LD/LD vs. DD/LD *p* > 0.9999, LD/LD vs. DD/DD *p* = 0.0159). Finally, *DD2R* showed the pattern of expression similar to *Dop1R2*, but the observed changes were not statistically significant (Figure 2D, LD/LD vs. DD/LD *p* = 0.0.6857, LD/LD vs. DD/DD *p* = 0.0635). The obtained results suggest more complex, cell-type specific regulation of dopamine signaling.

**FIGURE 3:**
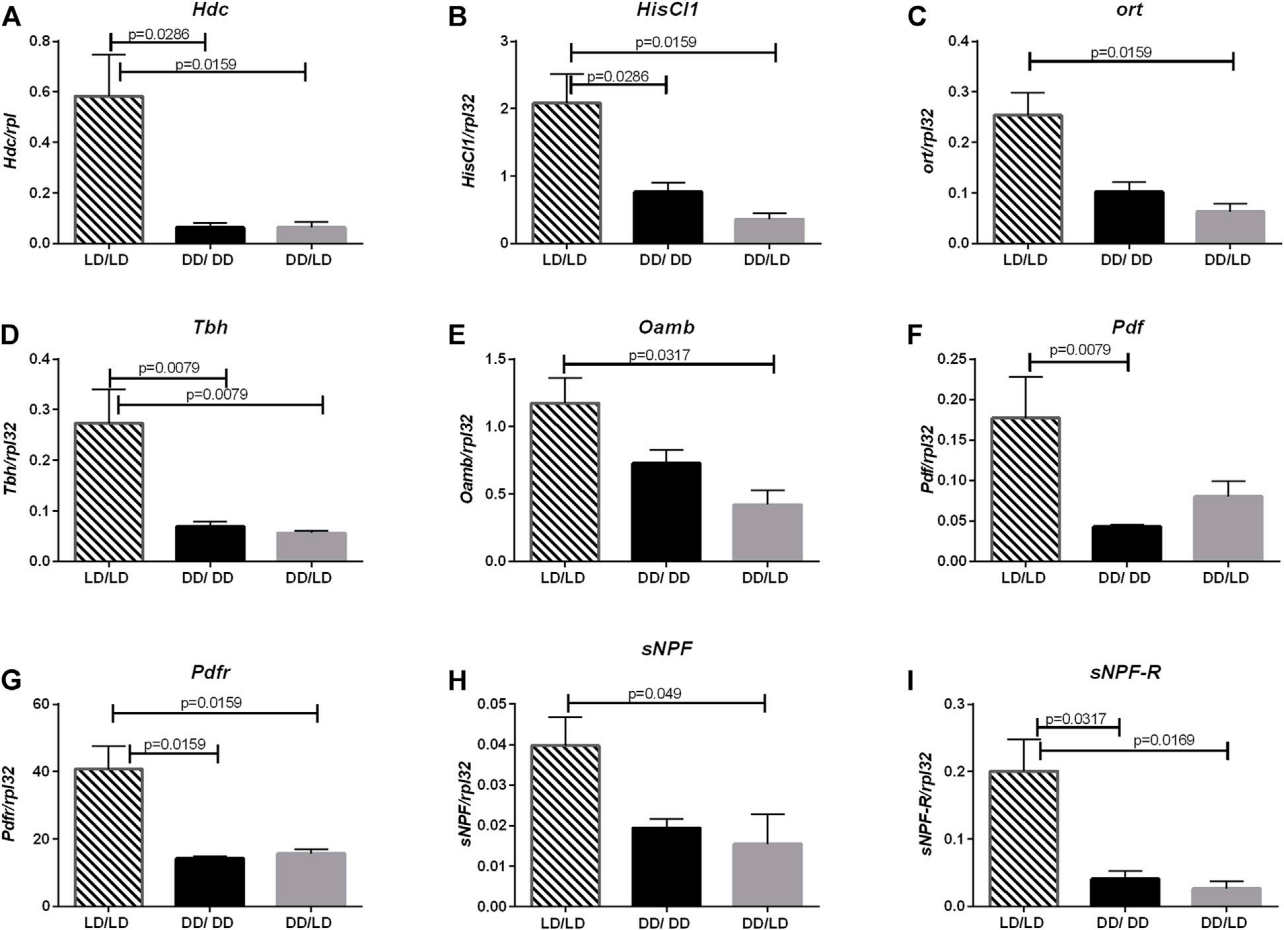
Lack of light changes neurotransmitters signaling level. qPCR analysis of expression level of genes connected with histamine, octopamine, PDF and sNPF signaling for flies maintained in normal conditions LD12:12 (LD/LD), maintained in DD and developed in darkness (DD/DD) and after eclosion transferred to LD12:12 (DD/LD). Expression checked for: **(A)** *histidine decarboxylase* (*Hdc*), **(B)** receptor *HisCl1*, **(C)** receptor *Ort.* **(D)** *Tbh* and **(E)** receptor *Oamb,* **(F)** *Pdf*, **(G)** *Pdfr*, **(H)** *sNPF* and **(I)** *sNPF-R. N* = 5. Mean ± SEM.

The another neurotransmitter involved in light transmission and sleep regulation is histamine. The expression pattern of *Hdc* (LD/LD vs. DD/LD *p* = 0.0079, LD/LD vs. DD/DD *p* = 0.0079), *HisCl1* (LD/LD vs. DD/LD *p* = 0.0159, LD/LD vs. DD/DD *p* = 0.0286) and *ort* (LD/LD vs. DD/LD *p* = 0.01159, LD/LD vs. DD/DD *p* = 0.0571) in our experiment was similar, with decreased level in flies kept in DD during development (Figures 3A–C).

The next pathway affected by light conditions during development was octopamine. Light during the development decreased the expression level of both, *Tbh* (LD/LD vs. DD/LD *p* = 0.0079, LD/LD vs. DD/DD *p* = 0.0079) and *Oamb* (LD/LD vs. DD/LD *p* = 0.0317, LD/LD vs. DD/DD *p* = 0.1143), however, flies developing in DD and then transferred to LD had the lowest amount of transcript (Figures 3D,E.).

Finally, we checked the expression level of two neurotransmitter genes known to be involved directly in the circadian control of sleep: sNPF and PDF and their receptors, sNPF-R and PDFR. All of them had the similar pattern—in flies developing in DD their expression level was decreased, and after transferring adults to LD, the level of mRNA did not change significantly (Figures 3F–I, for *Pdf* LD/LD vs. DD/LD *p* = 0.0952, LD/LD vs. DD/DD *p* = 0.0079, for *Pdfr* LD/LD vs. DD/LD *p* = 0.0159, LD/LD vs. DD/DD *p* = 0.0159, for *sNPF* LD/LD vs. DD/LD *p* = 0.049, LD/LD vs. DD/DD *p* = 0.0635, for *sNPF-R* LD/LD vs. DD/LD *p* = 0.0169, LD/LD vs. DD/DD *p* = 0.0317).

### Photoreception affects synthesis of neurotransmitters

**FIGURE 4:**
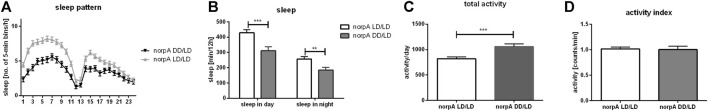
*NorpA* mutants show behavioral changes when developed without light. **(A)** Sleep pattern shows changes of sleep between experimental (DD/LD) and control group (LD/LD). **(B)** Sleep level is decreased both, during the day and night. **(C)** Total activity is significantly increased in flies which grown up in darkness. **(D)** Activity index measured as ratio of total activity per day and number of minutes awaked. Mean ± SEM. Statistically significant differences marked as asterisks: **p ≤ 0.01, ***p ≤ 0.001.

Flies receive light signal through different pathways. The most important is the retinal phototransduction pathway, which requires phospholipase C (PLC), encoded by the gene *norpA*. *NorpA* mutants are almost blind, however, they still have partial light sensitivity through deep brain photoreceptors. Because we did not observe significat changes in gene expression between wild type flies kept in DD/DD and DD/LD conditions, and to avoid impact of light input received by additional photoreceptors, we perform this experiment for LD/LD and DD/DD groups only. Flies with *norpA* mutation developing in LD12:12 conditions, showed normal activity profile and they were able to maintain rhythmicity in DD. However, similarly to CS, *norpA* mutants, developing in DD, were more active than control in LD12:12 (*p* = 0.0003), but activity index was not changed (*p* = 0.8724). Their pattern of sleep was changed and the total amount of sleep was decreased during the day (*p* = 0.0001) and night (*p* = 0.0039) (Figure 4).

Having in mind that photoreception through the retina photoreceptors can affect neurotransmission level in the brain we checked gene expression of the previously selected genes in head homogenates from *norpA* mutants.

**FIGURE 5:**
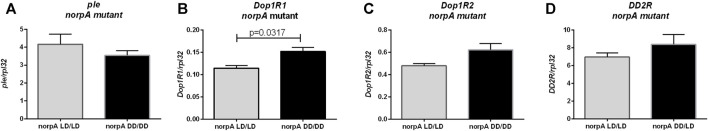
Light during early life affects only one of dopamine receptors in *norpA* mutant flies. qPCR analysis of expression level of genes connected with dopamine signaling for flies maintained in normal conditions LD12:12 (LD/LD), maintained and developed in darkness (DD/DD). Expression checked for: **(A)** *tyrosine hydroxylase* (*ple*), **(B)** dopamine receptor *Dop1R1*, **(C)** *Dop1R2* and **(D)** *DD2R*. *N* = 5. Mean ± SEM.

In dopamine pathway we found changes only in *Dop1R1* expression between *norpA* mutants developing in LD12:12 and DD (*p* = 0.0317), while tyrosine hydroxylase (*p* = 0.6905), Dop1R2 (*p* = 0.0952) and DD2R (*p* = 0.3095) encoding genes were expressed at the similar level in both groups (Figure 5). This result suggests that light during embryonic and postembryonic development affects *Dop1R2* and *ple* expression in PLC-dependent manner.

**FIGURE 6:**
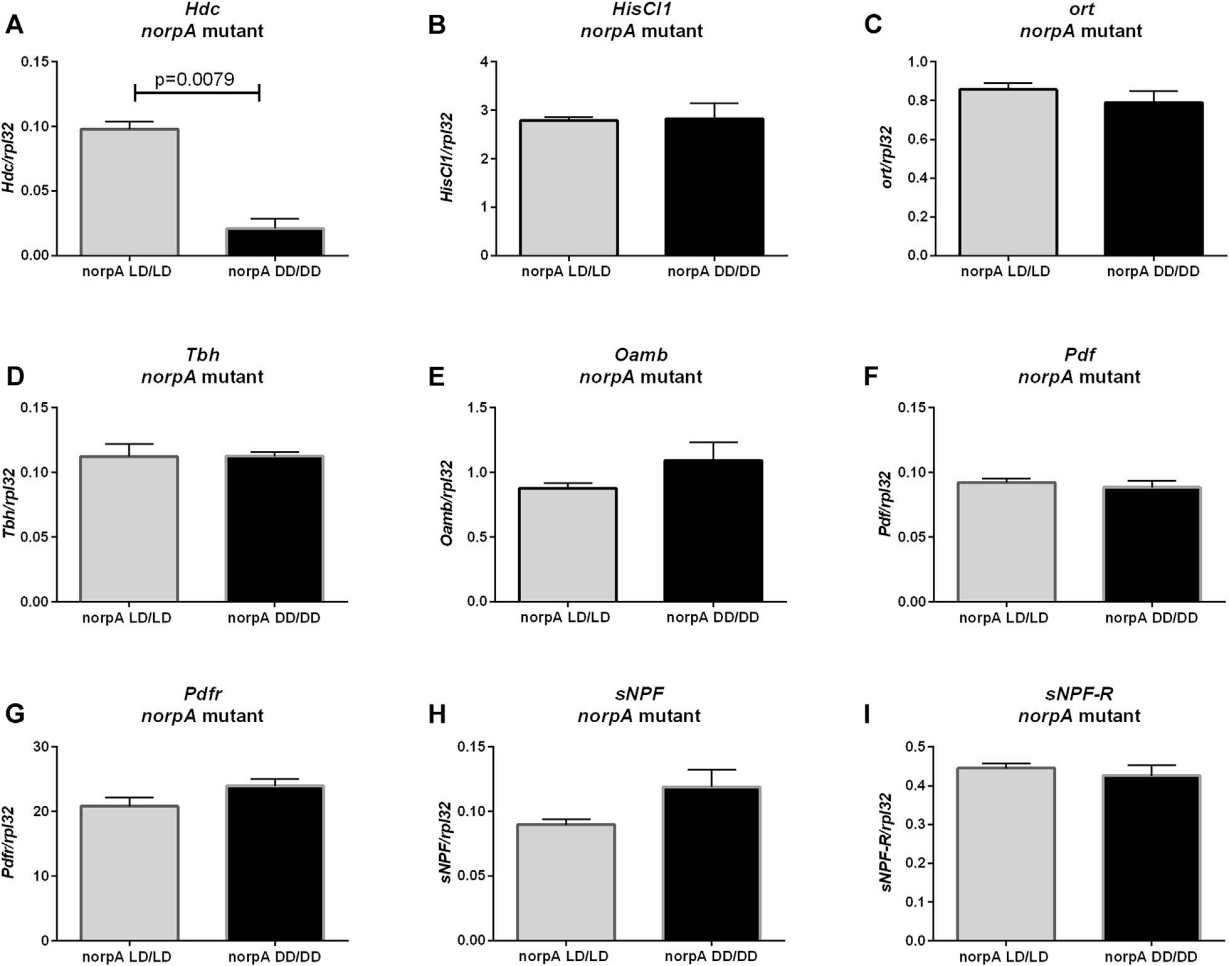
Effect of light on neurotransmitters pathway in *norpA* mutants. mRNA level was changed only for **(A)** *Hdc* gene. Histamine receptors: **(B)** *HisCl1* and **(C)** *ort* were expressed on the same level. Octopamine signaling seems to be not affected: **(D)** *Tbh*, **(E)** *Oamb*. Clock signaling was not changed: **(F)** *Pdf*, **(G)** *Pdfr*, **(H)** *sNPF*, **(I)** *sNPF-R*. *N* = 5. Mean ± SEM.

Histamine synthesis (*Hdc* gene expression) was decreased in *norpA* mutants growing without light (Figure 6A, *p* = 0.0079), but mRNA levels of *HisCl1* (Figure 6B, *p* = 0.8413) and *ort* genes of histamine receptors (Figure 6C, *p* = 0.4206) were unchanged. However, other neurotransmission pathways checked previously (octopamine, PDF and sNPF) seem do not to be PLC-dependent, as *Tbh* (Figure 6D, *p* > 0.9999)*, Oamb* (Figure 6E, *p* = 0.2222.), *Pdf* (Figure 6F, *p* = 0.8413)*, Pdfr* (Figure 6G, *p* = 0.1508)*, sNPF* (Figure 6H, *p* = 0.0556) and s*NPF-R* (Figure 6I, *p* > 0.9999) mRNAs had similar level in LD/LD and DD/DD groups in *norpA* mutants.

**FIGURE 7:**
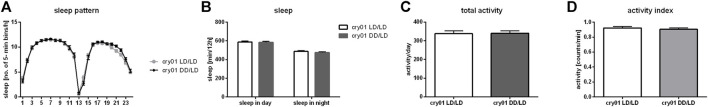
Cryptochrome is a key factor in larval light absorbance affecting behavior in adults. *cry* ^*01*^ mutants kept in darkness during development did not show changes in **(A)** sleep pattern, **(B)** sleep level nor **(C)** activity level. **(D)** Activity index measured as ratio of total activity per day and number of minutes awaked. Mean ± SEM.

To examine effect of light detected by deep brain photoreceptors, which can affect neurons directly, we used *cry* ^*01*^ mutants. Surprisingly, we did observe neither differences in activity (*p* = 0.9137), activity index (*p* = 0.5335), sleep pattern nor its level (for day *p* = 0.874, for night *p* = 0.334) between flies reared in LD and DD (Figure 7). This result indicates that CRY is the most important light photoreceptor during development.

**FIGURE 8:**
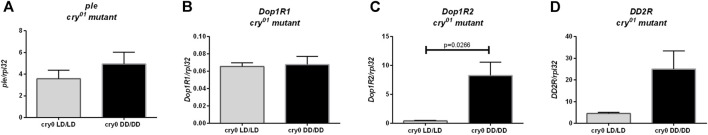
Light during early life affects only one of dopamine receptors in *cry* ^*01*^ mutant flies. qPCR analysis of expression level of genes connected with dopamine signaling for flies maintained in normal conditions LD12:12 (LD/LD), maintained in DD and developed in darkness (DD/DD). Expression checked for: **(A)** *tyrosine hydroxylase* (*ple*), **(B)** dopamine receptor *Dop1R1*, **(C)** *Dop1R2* and **(D)** *DD2R*. *N* = 5. Mean ± SEM.

**FIGURE 9:**
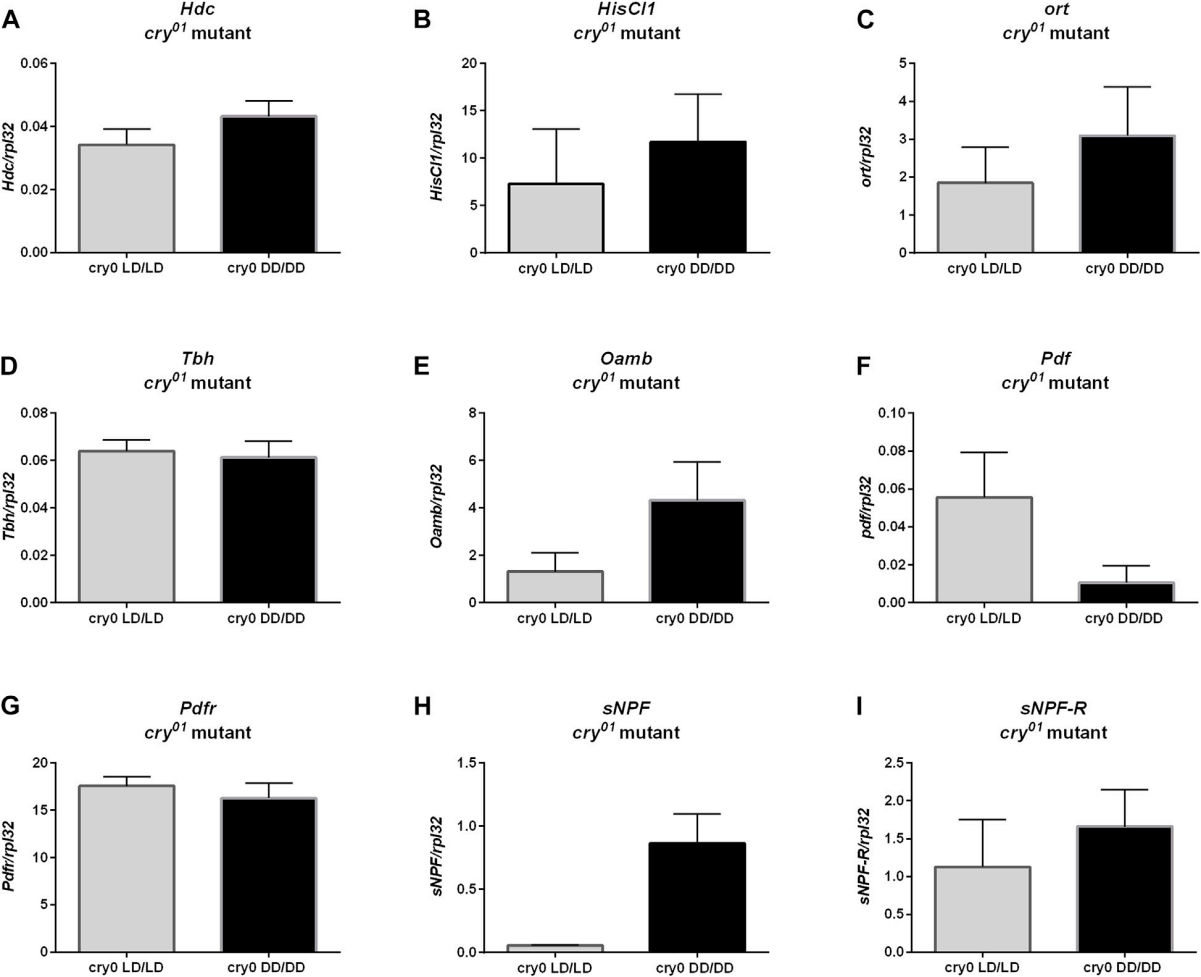
*cry* ^*01*^ mutants does not show changes in neurotransmitters signaling. mRNA of genes coding **(A)** *Hdc*, **(B)** *HisCl1*, **(C)** *ort*, **(D)** *Tbh*, **(E)** *Oamb* **(F)** *Pdf*, **(G)** *Pdfr*, **(H)** *sNPF* and **(I)** *sNPF-R* did not differ between groups maintained in LD12:12 and darkness during early life. *N* = 5. Mean ± SEM.

Moreover, we did not observe changes in *ple* (Figure 8A, *p* = 0.4), *Dop1R1* (Figure 8B, *p* > 0.9999) nor *DD2R* expression (Figure 8D, *p* = 0.0571), but *Dop1R2* mRNA level was increased in flies reared in DD, similarly to wild-type flies (Figure 8C, *p* = 0.0266). Histamine (*Hdc*, Figure 9A, *p* = 0.309, *HisCl1*, Figure 9B, *p* = 0.4, *Ort*, Figure 9C, *p* = 0.2857), octopamine (*Tbh*, Figure 9D, *p* = 0.8413, *Oamb*, Figure 9E, *p* = 0.1143), PDF (*Pdf*, Figure 9F, *p* = 0.3095, *Pdfr*, Figure 9G, *p* = 0.5476) and sNPF (*sNPF*, Figure 9H, *p* = 0.0571, *sNPF-R*, Figure 9I, *p* = 0.5476) signaling components were also not changed.

### Light affects development of mushroom bodies and dopaminergic input to mushroom bodies

**FIGURE 10:**
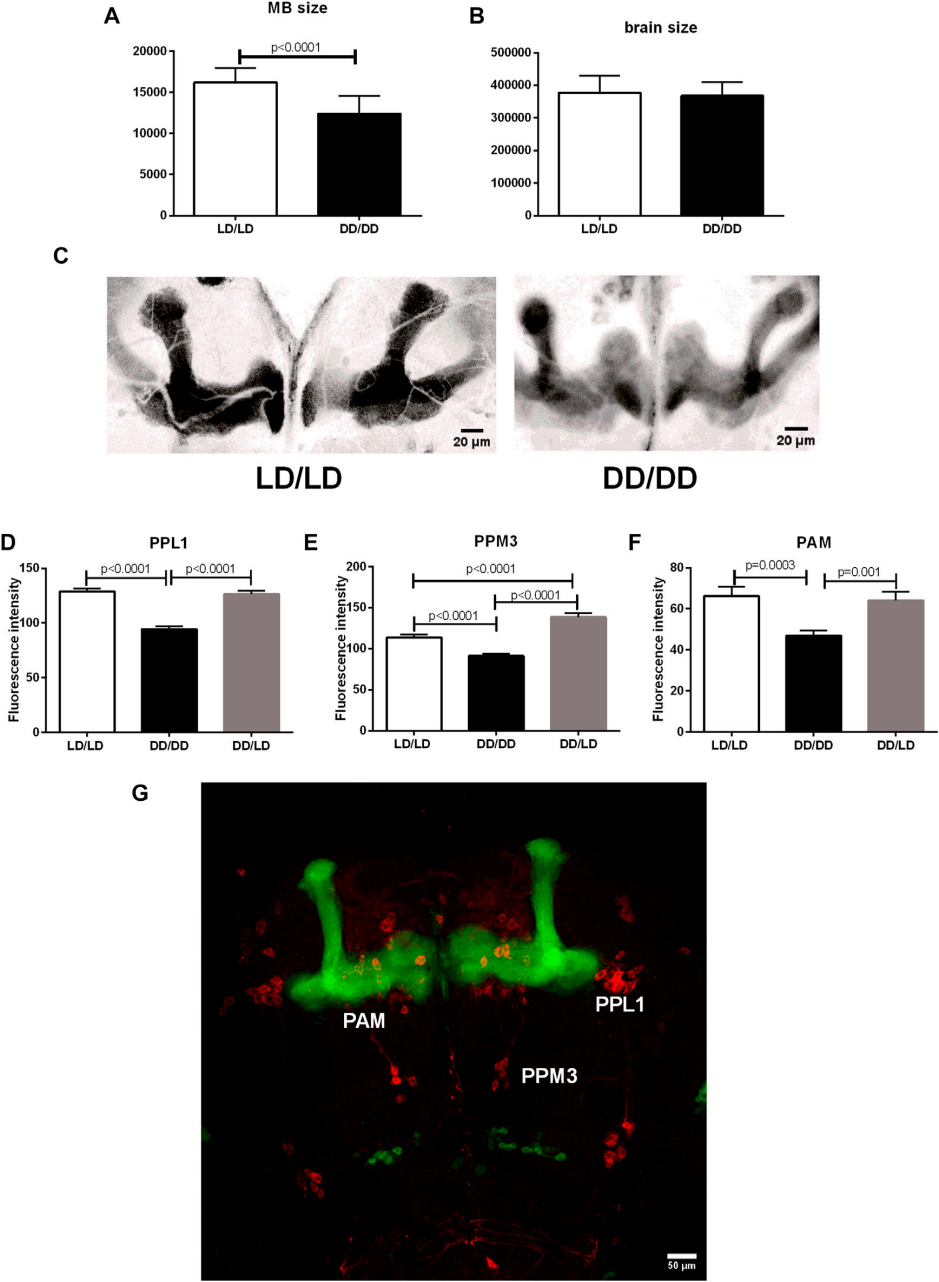
Mushroom bodies development and its dopaminergic input depends on light during early life. **(A)** The size of MBs is significantly decreased in flies from DD/DD group, while **(B)** the whole brain size was not changed. **(C)** Representative confocal images of MB from flies developed in LD and DD conditions. Images showed here were modified: selected area was cropped, 8-bit image was color inverted and contrast was enhanced. **(D–G)**: Tyrosine hydroxylase immunofluorescence signal measured in specific dopaminergic cell clusters innervating sleep centers showed decreased level in DD/DD group. DD/LD group showed TH level similar to the control. Total number of brains used for experiments: 30 per group. Mean ± SEM. **(G)**: confocal image of the brain - OK107 > GFP strain has visible mushroom bodies (green), anti-TH immunostaining (red) marks dopaminergic clusters.

Sleep is controlled by many different cell types, but the most important ones are sleep centers located in the mushroom bodies (MB). Because we observed the strong effect of light during development on sleep level in adult flies, we wondered whether it can be correlated with MB size changes. We used transgenic cross OK-107-Gal4 with UAS-mCD8::GFP to visualize MB. We reared flies in LD or DD, respectively and collected brains of 2-days old males. We measured brain size as well as mushroom bodies of every single individual and found that flies kept in DD had smaller MB (*p* < 0.0001), while the brain size was not changed (*p* = 0.4483) (Figures 10A–C).

Additionally, we checked tyrosine hydroxylase immunofluorescence in specific cell clusters innervating MB. We found that flies kept in DD during development have lower expression of TH in PPL1, PPM3 and PAM neurons, however, transferring flies to the light:dark conditions restored normal level of the enzyme, but in the case of PPM3 the amount of TH was higher than in the control group (Figures 10D–G).

## Discussion

Sleep is one of most important functions of organism. During resting time many processes occur, like memory consolidation, wound healing, etc. Sleep is regulated by internal signals coming from the circadian clock and sleep centers, but it is also responsive to external signals, mostly light inputs. Sleep regulation is very complex and requires multicellular communication and homeostasis. Light is a well-known arousal factor, however, mid-day siesta is promoted during the most intense light exposure.

Mechanisms affecting sleep have already been investigated carefully focusing on different aspects, however, little is known about the role of light during development in the regulation of sleep. Larvae have the circadian clock, which can be synchronized by environmental light. Adults eclosing from pupae, which during postembryonic development were reared in constant light or constant darkness, can be easily synchronized by light and they are able to keep normal period of locomotor activity in DD (Sheeba et al., 2002; Zhao et al., 2019). Moreover, single 12 h light pulse in the first instar larva is sufficient to keep the daily rhythmicity in adult life. It has been shown that this entrainment can be observed in an individual fly, however, the whole population is not synchronized, meaning that each fly has different phase of the rhythm. This indicates that the endogenous clock starts to tick independently of light and goes continuously until adulthood (Sehgal et al., 1992; Zhao et al., 2019). The larval time-memory, results from cyclic *per* and *tim* expression in the pacemaker in the brain (Kaneko et al., 1997). However, in peripheral oscillators PER is expressed during embryogenesis and postembryonic development, and its level increases during development, but there are no daily cycling of PER before adulthood (Zhao et al., 2019). A light pulse given to larvae causes phase shift of the rhythm in adults and there are differences in the magnitude and direction of shifts between wild type and *per* ^*s*^ mutants (Kaneko et al., 2000). The mechanism of this process depends on changes in the larval molecular clock induced by light received by both photoreceptive pathways: CRY- and rhodopsin-dependent (Kaneko et al., 2000). Having in mind these larval time-memory aspects, it was not surprising that flies used in our experiments showed normal pattern of activity in adult life and they were able to keep rhythmicity in constant darkness with the period length similar to control. However, total level of activity was increased in insects reared in DD, and their activity index was higher, which means that they were more active during waking time. They also slept less, especially during the night when compared to flies developing in normal LD12:12 conditions. Interestingly, sleep was decreased during first 8 h of the day and during the night starting 3 h after lights-off, while during the rest of the time, the number of sleep bins was similar to the control group. Flies were already synchronized to LD12:12 regime, as morning and evening peaks of activity, amplitude of the rhythm and time of sleep were the same in both groups. Similar changes, higher activity and decreased sleep time, were observed in *norpA* mutants, however, activity index was not changed and sleep was affected in both during the day and night. This indicates that light perception through photoreceptors is only partially responsible for the observed changes in this behavior. On the other hand, flies lacking functional CRY did not show any changes in activity and sleep profile and level, which indicates that CRY is a key factor affecting observed changes.

The obtained results suggest that our experimental flies had the normally working circadian clock, but some pathways regulating wakefulness were enhanced. Our first goal was to check dopamine, as it is well-known wake-promoting factor (Andretic et al., 2005). Surprisingly, wild-type flies reared in DD showed reduced expression level of *ple,* encoding tyrosine hydroxylase (TH)—key enzyme in dopamine synthesis, and in effect also dopamine level in the brain most probably was decreased. Since the inhibition of dopamine-releasing neurons causes hyperactivity in response to mechanical startle (Friggi-Grelin et al., 2003), it is possible that also our experimental flies having lower dopamine level could be more active after light changes, as we observed most severe changes in behavior in hours after lights-on and lights-off. Moreover, our flies were probably stressed by transferring them from DD to LD12:12, so they could be also more sensitive to light, and all of this can reduce dopamine level (Neckameyer and Weinstein, 2005). On the other hand, the group DD/DD had lower *ple* expression level, even without any light input. To investigate this problem in details, we focused on dopaminergic neurons innervating the central complex (PPM3) and mushroom bodies (PPL1 and PAM), the structures hosting a sleep center (Mao and Davis, 2009). Immunofluorescence intensity measurements showed that the lack of light during development decreased TH level in these clusters, however transferring flies to LD rescued this effect. It means that light can directly enhance dopamine synthesis in specific cells, which can affect sleep control. Dysregulation of the balance in dopamine production between different clusters may enhance stress and in effect promote wakefulness.

Although our experimental flies showed decreased dopamine synthesis, at the same time they had increased *Dop1R1* and *Dop1R2* expression. This support our findings that dopamine signaling is changed in specific target neurons. *DopR* mutants showed increased sleep level (Lebestky et al., 2009), which supports our data, that shorter sleep time is correlated with increased *DopR* expression. However, DopR regulates Repetitive Startle-induced Hyperactivity (ReSH) and sleep in the opposite way, acting on specific cells (Lebestky et al., 2009). Wake-promoting effect of dopamine, but not ReSH, occurs mostly in clock neurons (Parisky et al., 2008; Shang et al., 2008; Lebestky et al., 2009). The main pacemaker cells, l-LNvs, are wake-promoting neurons during the day (Parisky et al., 2008; Shang et al., 2008), however, they do not play this role in constant darkness (Shang et al., 2008), as their firing rate is enhanced by the acute light exposure (Sheeba et al., 2008a; 2008b). This group of cells is sensitive to dopamine, which increases cAMP level and causes depolarization of cell membrane, but it does not affect firing rates of the l-LNvs (Shang et al., 2011; Fernandez-Chiappe et al., 2020). Moreover, decreasing of Dop1R2 expression in s-LNvs decreases nighttime sleep (Fernandez-Chiappe et al., 2020). Dopamine signaling to l-LNvs is suppressed by light, and in effect, sleep during the night is more sensitive to dopamine than during the day, and this effect is even stronger in constant darkness (Shang et al., 2011). Our data suggest that *Dop1R2* expression can be inhibited by light directly, as flies developing and maintained continuously in DD showed higher level of *Dop1R2* mRNA than flies reared in DD and transferred to LD (DD/LD group) or the control flies developing in LD12:12 (LD/LD group). On the other hand, *ple* and *Dop1R1* expression seems to be continuously inhibited by the lack of light during development, while *DD2R* expression level is not affected by light conditions. The results obtained for *cry* ^*01*^ and *norpA* mutants are very interesting, since they show that gene expression of the dopamine pathway in the group DD/LD strictly depends on photoreception—*Dop1R1* is CRY-dependent, while *Dop1R2* is PLC—dependent, and *ple* is both CRY and PLC dependent. The fact, that dopamine acts through Cryptochrome, and both of them are required for nocturnal increased activity was shown also by other authors (Kumar et al., 2012). However, only Dop1R1 seems to be related to the disruption of night-time sleep in flies from DD/DD group, as its enhanced expression follows behavioral changes in wild-type and *norpA* mutants, while *cry* ^*01*^ kept in the same light regimes does not change behavior nor Dop1R1 level.

In case of histamine, the main neurotransmitter used by retina photoreceptors, it has been shown that it is involved in the regulation of sleep/wake cycles through HisCl1 located on clock neurons (Oh et al., 2013; Alejevski et al., 2019). Flies maintained in DD during development had lower expression of *Hdc* gene, which encodes a key enzyme in histamine synthesis. Moreover, gene expression of both known receptors HisCl1 and Ort was decreased. Histamine has been identified as a wake-promoting agent however our experimental flies showed decreased histamine and increased both activity time and activity during waking. Light effect on histamine synthesis seems to be precisely regulated by Cryptochrome, but not by PLC, while expression of *HisCl1* and *ort* receptor genes are regulated by both PLC and CRY.

Another neurotransmission pathway involved in sleep and affected by darkness during the postembryonic development was octopamine. Here, again, in flies reared in DD we observed lower expression of *Tbh* (a key enzyme involved in octopamine synthesis) and octopamine receptor gene *Oamb*, which indicates that this signaling pathway was decreased in adults. It was previously shown that octopamine regulates activity/sleep cycle in larvae, since the activation of Tdc-expressing neurons decreases sleep time, while *Tbh* and *Oamb* mutations or silencing increases sleep time (Szuperak et al., 2018). Octopamine is also a wake-promoting agent in adult flies, which forces l-LN_v_ cells to release PDF during the day (Oh et al., 2014). However, it has been shown that sleep regulation using this neurotransmitter does not involve mushroom bodies, although they express octopamine receptors (Crocker and Sehgal, 2008). Octopamine signaling inhibition in DD applied during development, seems to depend on both CRY and PLC, because it was not changed in *cry* ^*0*1^ and *norpA* mutants. However, our results suggest that the observed changes in mRNA levels of *cry* ^*0*1^ and *norpA* do not drive sleep inhibition during the night.

Neurotransmitters involved in the regulation of the clock mechanism are very interesting in the context of sleep and light. The main clock neurotransmitter is PDF and its function in sleep regulation is very complex. It inhibits dopaminergic arousal neurons PPM3 or activates DN1p, which in turn inhibits tubular bulbar cells (Tubu). PDF released from l-LNVs is also responsible for nocturnal activity (Sheeba et al., 2008a) and light-dependent arousal (Shang et al., 2008). It is possible that the lack of light during development causes defects in the l-LNv excitability, which in turn affects cell physiology, neurotransmitter production, and in effect sleep time. This is supported by the fact, that light-induced firing-rate of l-LNvs depends on CRY, and is not observed in *cry* mutants (Emery et al., 2000; Sheeba et al., 2008b; Fogle et al., 2011). Decreased *Pdf* and *Pdfr* expression observed in our experimental flies may cause weaker inhibition of PPM3 and, in effect, higher total activity.

The second neurotransmitter present in the circadian clock neurons, called sNPF is broadly expressed in the brain, including sleep centers (Nässel et al., 2008; Johard et al., 2009) and its role in the sleep regulation is well documented (Shang et al., 2013). sNPF expressed in s-LNvs shows circadian changes in abundance and promotes normal night-time sleep (Kula-Eversole et al., 2010; Shang et al., 2013), which matches with our results. We observed that expression of PDF and sNPF, as well as their receptors is regulated by light during development in PLC and CRY-dependent manner. However, these changes do not affect behavior.

Comparing wild-type flies with mutants used in the study we can conclude that the exposure to DD during development affects sleep during the night, reduces its time and increases activity time, with lower locomotor activity during wake. This indicates that experimental flies spent more time being active, but their activity level was similar to that in the control. Observed changes are regulated mostly by increasing *DopR1* expression. Changes in the expression of other gene studied seems do not to be connected with the observed behavioral phenotype because we did not observe them in *norpA* mutants which show nocturnal sleep deficits similar to wild-type flies. However, *norpA* mutants showed increased total activity time with no changes of activity index, which indicates that they were more and longer active than blind flies reared in LD.

The last part of our work showed that lack of light during the development affects the size of mushroom bodies. Flies kept in DD had similar body size, their brain size was also not changed, but the area of MB was significantly decreased. It was previously shown that larvae in DD have reduced number of ganglion mother cells (GMCs) derived from mushroom bodies neuroblasts, similarly to *per* ^*01*^ and *cry* ^*01*^ mutants kept in LD12:12 (Dapergola et al., 2021). In addition, NBs size was smaller than in control, and their transcriptional activity was decreased. In effect, the size of larval brain was decreased too. Surprisingly, the size of adult brain was normal (Dapergola et al., 2021), which was also confirmed in our study. As it was shown that development of mushroom bodies is affected by light conditions, we decided to check what happens with this sleep-controlling structure in the adult brain. Our results showed that the MB development was constantly affected, as in DD they did not grow to the normal size. It supports an idea that light is necessary for proliferation of specific cell types in the brain. However, since we did not measure Kenyon cells number, it is possible that size changes are the consequence of decreased number of Kc cells or their processes and arborizations, which build MB lobes. Because the total size of the brain was not changed, other brain areas should be enlarged, however we did not have methods to check this interesting issue. The question which part of the brain or which cell types can compensate this difference in size reminds open for further investigations. What is interesting, sleep deprivation also affects NB number (Szuperak et al., 2018). It has been shown that 3 h of mechanical sleep deprivation in the second instar larvae inhibits neuroblasts proliferation in about 15%, and this effect can be recovered after sleep rebound (Szuperak et al., 2018). One of the explanations of the smaller size of MB in flies developing in DD might be that, lack of light decreases their sleep time during larval stage, which in turn affects NBs proliferation rate. This indicates how important is light, clock and sleep in early stages of brain development.

## Data Availability

The original contributions presented in the study are included in the article/supplementary materials, further inquiries can be directed to the corresponding author.

## References

[B1] Alejevski F. Saint-Charles A. Michard-Vanhée C. Martin B. Galant S. Vasiliauskas D. (2019). The HisCl1 histamine receptor acts in photoreceptors to synchronize Drosophila behavioral rhythms with light-dark cycles. Nat. Commun. 10, 252. 10.1038/s41467-018-08116-7 30651542 PMC6335465

[B2] Andretic R. Van Swinderen B. Greenspan R. J. (2005). Dopaminergic modulation of arousal in Drosophila. Curr. Biol. 15, 1165–1175. 10.1016/j.cub.2005.05.025 16005288

[B3] Aso Y. Hattori D. Yu Y. Johnston R. M. Nirmala A. Ngo T. (2014). The neuronal architecture of the mushroom body provides a logic for associative learning. eLife 3, e04577. 10.7554/eLife.04577 25535793 PMC4273437

[B4] Buchner E. Buchner S. Crawford G. Mason W. T. Salvaterra P. M. Sattelle D. B. (1986). Choline acetyltransferase-like immunoreactivity in the brain of *Drosophila melanogaster* . Cell Tissue Res. 246. 10.1007/BF00218999

[B5] Busto M. Iyengar B. Campos A. R. (1999). Genetic dissection of behavior: Modulation of locomotion by light in the *Drosophila melanogaster* larva requires genetically distinct visual system functions. J. Neurosci. 19, 3337–3344. 10.1523/jneurosci.19-09-03337.1999 10212293 PMC6782248

[B6] Chatterjee A. Lamaze A. De J. Mena W. Chélot E. Martin B. (2018). Reconfiguration of a multi-oscillator network by light in the Drosophila circadian clock. Curr. Biol. 28, 2007–2017. e4. 10.1016/j.cub.2018.04.064 29910074 PMC6039274

[B7] Chen K. F. Peschel N. Zavodska R. Sehadova H. Stanewsky R. (2011). QUASIMODO, a novel GPI-anchored Zona Pellucida protein involved in light input to the drosophila circadian clock. Curr. Biol. 21, 719–729. 10.1016/j.cub.2011.03.049 21530261

[B8] Chung B. Y. Kilman V. L. Keath J. R. Pitman J. L. Allada R. (2009). The GABAA receptor RDL acts in peptidergic PDF neurons to promote sleep in Drosophila. Curr. Biol. 19, 386–390. 10.1016/j.cub.2009.01.040 19230663 PMC3209479

[B9] Crocker A. Sehgal A. (2008). Octopamine regulates sleep in Drosophila through protein kinase A-dependent mechanisms. J. Neurosci. 28, 9377–9385. 10.1523/JNEUROSCI.3072-08a.2008 18799671 PMC2742176

[B10] Crocker A. Shahidullah M. Levitan I. B. Sehgal A. (2010). Identification of a neural circuit that underlies the effects of octopamine on sleep:wake behavior. Neuron 65, 670–681. 10.1016/j.neuron.2010.01.032 20223202 PMC2862355

[B11] Cusumano P. Klarsfeld A. Chélot E. Picot M. Richier B. Rouyer F. (2009). PDF-modulated visual inputs and cryptochrome define diurnal behavior in Drosophila. Nat. Neurosci. 12, 1431–1437. 10.1038/nn.2429 19820704

[B12] Damulewicz M. Ispizua J. I. Ceriani M. F. Pyza E. M. (2020). Communication among photoreceptors and the central clock affects sleep profile. Front. Physiol. 11, 993–1017. 10.3389/fphys.2020.00993 32848895 PMC7431659

[B13] Dapergola E. Menegazzi P. Raabe T. Hovhanyan A. (2021). Light stimuli and circadian clock affect neural development in *Drosophila melanogaster* . Front. Cell Dev. Biol. 9, 595754–595814. 10.3389/fcell.2021.595754 33763414 PMC7982892

[B14] Dolezelova E. Dolezel D. Hall J. C. (2007). Rhythm defects caused by newly engineered null mutations in Drosophila’s cryptochrome gene. Genetics 177, 329–345. 10.1534/genetics.107.076513 17720919 PMC2013679

[B15] Donlea J. M. Thimgan M. S. Suzuki Y. Gottschalk L. Shaw P. J. (2011). Inducing sleep by remote control facilitates memory consolidation in Drosophila. Science 332, 1571–1576. 10.1126/science.1202249 21700877 PMC4064462

[B16] Emery P. Stanewsky R. Hall J. C. Rosbash M. (2000). A unique circadian-rhythm photoreceptor. Nature 404, 456–457. 10.1038/35006558 10761904

[B17] Fernandez-Chiappe F. Hermann-Luibl C. Peteranderl A. Reinhard N. Senthilan P. R. Hieke M. (2020). Dopamine signaling in wake-promoting clock neurons is not required for the normal regulation of sleep in drosophila. J. Neurosci. 40, 9617–9633. 10.1523/JNEUROSCI.1488-20.2020 33172977 PMC7726529

[B18] Fogle K. J. Parson K. G. Dahm N. A. Holmes T. C. Sheeba V. Parisky K. M. (2011). CRYPTOCHROME is a blue-light sensor that regulates neuronal firing rate. Science 331, 1409–1413. 10.1126/science.1199702 21385718 PMC4418525

[B19] Friggi-Grelin F. Coulom H. Meller M. Gomez D. Hirsh J. Birman S. (2003). Targeted gene expression in Drosophila dopaminergic cells using regulatory sequences from tyrosine hydroxylase. J. Neurobiol. 54, 618–627. 10.1002/neu.10185 12555273

[B20] Grima B. Chélot E. Xia R. Rouyer F. (2004). Morning and evening peaks of activity rely on different clock neurons of the Drosophila brain. Nature 431, 869–873. 10.1038/nature02935 15483616

[B21] Guo F. Holla M. Díaz M. M. Rosbash M. (2018). A circadian output circuit controls sleep-wake arousal in Drosophila. Neuron 100, 624–635. 10.1016/j.neuron.2018.09.002 30269992

[B22] Guo F. Yu J. Jung H. J. Abruzzi K. C. Luo W. Griffith L. C. (2016). Circadian neuron feedback controls the Drosophila sleep-activity profile. Nature 536, 292–297. 10.1038/nature19097 27479324 PMC5247284

[B23] Hamasaka Y. Nässel D. R. (2006). Mapping of serotonin, dopamine, and histamine in relation to different clock neurons in the brain of Drosophila. J. Comp. Neurol. 494, 314–330. 10.1002/cne.20807 16320241

[B24] Hassan J. Busto M. Iyengar B. Campos A. R. (2000). Behavioral characterization and genetic analysis of the *Drosophila melanogaster* larval response to light as revealed by a novel individual assay. Behav. Genet. 30, 59–69. 10.1023/A:1002090627601 10934800

[B25] Haynes P. R. Christmann B. L. Griffith L. C. (2015). A single pair of neurons links sleep to memory consolidation in *Drosophila melanogaster* . Elife 4. 10.7554/eLife.03868 PMC4305081 25564731

[B26] Helfrich-Förster C. Edwards T. Yasuyama K. Wisotzki B. Schneuwly S. Stanewsky R. (2002). The extraretinal eyelet of Drosophila: Development, ultrastructure, and putative circadian function. J. Neurosci. 22, 9255–9266. 10.1523/jneurosci.22-21-09255.2002 12417651 PMC6758046

[B27] Hendricks J. C. Finn S. M. Panckeri K. A. Chavkin J. Williams J. A. Sehgal A. (2000). Rest in Drosophila is a sleep-like state. Neuron 25, 129–138. 10.1016/S0896-6273(00)80877-6 10707978

[B28] Huber R. Hill S. L. Holladay C. Biesiadecki M. Tononi G. Cirelli C. (2004). Sleep homeostasis in *Drosophila melanogaster* . Sleep 27, 628–639. 10.1093/sleep/27.4.628 15282997

[B29] Johard H. A. D. Yoishii T. Dircksen H. Cusumano P. Rouyer F. Helfrich-Förster C. (2009). Peptidergic clock neurons in Drosophila: Ion transport peptide and short neuropeptide F in subsets of dorsal and ventral lateral neurons. J. Comp. Neurol. 516, 59–73. 10.1002/cne.22099 19565664

[B30] Joiner W. J. Crocker A. White B. H. Sehgal A. (2006). Sleep in Drosophila is regulated by adult mushroom bodies. Nature 441, 757–760. 10.1038/nature04811 16760980

[B31] Kaneko M. Hamblen M. J. Hall J. C. (2000). Involvement of the period gene in developmental time-memory: Effect of the per(Short) mutation on phase shifts induced by light pulses delivered to Drosophila larvae. J. Biol. Rhythms 15, 13–30. 10.1177/074873040001500103 10677013

[B32] Kaneko M. Helfrich-Förster C. Hall J. C. (1997). Spatial and temporal expression of the period and timeless genes in the developing nervous system of drosophila: Newly identified pacemaker candidates and novel features of clock gene product cycling. J. Neurosci. 17, 6745–6760. 10.1523/jneurosci.17-17-06745.1997 9254686 PMC6573141

[B33] Keene A. C. Mazzoni E. O. Zhen J. Younger M. A. Yamaguchi S. Blau J. (2011). Distinct visual pathways mediate drosophila larval light avoidance and circadian clock entrainment. J. Neurosci. 31, 6527–6534. 10.1523/JNEUROSCI.6165-10.2011 21525293 PMC3103866

[B34] Kistenpfennig C. Grebler R. Ogueta M. Hermann-Luibl C. Schlichting M. Stanewsky R. (2017). A new rhodopsin influences light-dependent daily activity patterns of fruit flies. J. Biol. Rhythms 32, 406–422. 10.1177/0748730417721826 28840790

[B35] Klarsfeld A. Picot M. Vias C. Chélot E. Rouyer F. (2011). Identifying specific light inputs for each subgroup of brain clock neurons in Drosophila larvae. J. Neurosci. 31, 17406–17415. 10.1523/JNEUROSCI.5159-10.2011 22131402 PMC6623821

[B36] Kula-Eversole E. Nagoshi E. Shang Y. Rodriguez J. Allada R. Rosbash M. (2010). Surprising gene expression patterns within and between PDF-containing circadian neurons in Drosophila. Proc. Natl. Acad. Sci. U. S. A. 107, 13497–13502. 10.1073/pnas.1002081107 20624977 PMC2922133

[B37] Kumar S. Chen D. Sehgal A. (2012). Dopamine acts through Cryptochrome to promote acute arousal in Drosophila. Genes Dev. 26, 1224–1234. 10.1101/gad.186338.111 22581798 PMC3371410

[B38] Kunst M. Hughes M. E. Raccuglia D. Felix M. Li M. Barnett G. (2014). Calcitonin gene-related peptide neurons mediate sleep-specific circadian output in Drosophila. Curr. Biol. 24, 2652–2664. 10.1016/j.cub.2014.09.077 25455031 PMC4255360

[B39] Lebestky T. Chang J. S. C. Dankert H. Zelnik L. Kim Y. C. Han K. A. (2009). Two different forms of arousal in Drosophila are oppositely regulated by the dopamine D1 receptor ortholog DopR via distinct neural circuits. Neuron 64, 522–536. 10.1016/j.neuron.2009.09.031 19945394 PMC2908595

[B40] Li J. Mahoney B. D. Jacob M. S. Caron S. J. C. (2020). Visual input into the *Drosophila melanogaster* mushroom body. Cell Rep. 32, 108138. 10.1016/j.celrep.2020.108138 32937130 PMC8252886

[B41] Liu Q. Liu S. Kodama L. Driscoll M. R. Wu M. N. (2012). Two dopaminergic neurons signal to the dorsal fan-shaped body to promote wakefulness in Drosophila. Curr. Biol. 22, 2114–2123. 10.1016/j.cub.2012.09.008 23022067 PMC3505250

[B42] Liu W. W. Wilson R. I. (2013). Glutamate is an inhibitory neurotransmitter in the Drosophila olfactory system. Proc. Natl. Acad. Sci. U. S. A. 110, 10294–10299. 10.1073/pnas.1220560110 23729809 PMC3690841

[B43] Malpel S. Klarsfeld A. Rouyer F. (2002). Larval optic nerve and adult extra-retinal photoreceptors sequentially associate with clock neurons during Drosophila brain development. Development 129, 1443–1453. 10.1242/dev.129.6.1443 11880353

[B44] Mao Z. Davis R. L. (2009). Eight different types of dopaminergic neurons innervate the Drosophila mushroom body neuropil: Anatomical and physiological heterogeneity. Front. Neural Circuits 3, 5. 10.3389/neuro.04.005.2009 19597562 PMC2708966

[B45] Mauss A. S. Meier M. Serbe E. Borst A. (2014). Optogenetic and pharmacologic dissection of feedforward inhibition in Drosophila motion vision. J. Neurosci. 34, 2254–2263. 10.1523/JNEUROSCI.3938-13.2014 24501364 PMC6608528

[B46] Mauss A. S. Pankova K. Arenz A. Nern A. Rubin G. M. Borst A. (2015). Neural circuit to integrate opposing motions in the visual field. Cell 162, 351–362. 10.1016/j.cell.2015.06.035 26186189

[B47] Mazzoni E. O. Desplan C. Blau J. (2005). Circadian pacemaker neurons transmit and modulate visual information to control a rapid behavioral response. Neuron 45, 293–300. 10.1016/j.neuron.2004.12.038 15664180

[B48] McCarthy E. V. Wu Y. deCarvalho T. Brandt C. Cao G. Nitabach M. N. (2011). Synchronized bilateral synaptic inputs to *Drosophila melanogaster* neuropeptidergic rest/arousal neurons. J. Neurosci. 31, 8181–8193. 10.1523/JNEUROSCI.2017-10.2011 21632940 PMC3125135

[B49] Morante J. Desplan C. (2008). The color-vision circuit in the medulla of Drosophila. Curr. Biol. 18, 553–565. 10.1016/j.cub.2008.02.075 18403201 PMC2430089

[B50] Nässel D. R. Enell L. E. Santos J. G. Wegener C. Johard H. A. D. (2008). A large population of diverse neurons in the Drosophila central nervous system expresses short neuropeptide F, suggesting multiple distributed peptide functions. BMC Neurosci. 9, 90. 10.1186/1471-2202-9-90 18803813 PMC2569041

[B51] Neckameyer W. S. Weinstein J. S. (2005). Stress affects dopaminergic signaling pathways in *Drosophila melanogaster* . Stress 8, 117–131. 10.1080/10253890500147381 16019603

[B52] Oh Y. Jang D. Sonn J. Y. Choe J. (2013). Histamine-HisCl1 receptor Axis regulates wake-promoting signals in *Drosophila melanogaster* . PLoS One 8, e68269. 10.1371/journal.pone.0068269 23844178 PMC3700972

[B53] Oh Y. Yoon S. E. Zhang Q. Chae H. S. Daubnerová I. Shafer O. T. (2014). A homeostatic sleep-stabilizing pathway in Drosophila composed of the sex peptide receptor and its ligand, the myoinhibitory peptide. PLoS Biol. 12, e1001974. 10.1371/journal.pbio.1001974 25333796 PMC4204809

[B54] Pantazis A. Segaran A. Liu C. H. Nikolaev A. Rister J. Thum A. S. (2008). Distinct roles for two histamine receptors (hclA and hclB) at the Drosophila photoreceptor synapse. J. Neurosci. 28, 7250–7259. 10.1523/JNEUROSCI.1654-08.2008 18632929 PMC6670387

[B55] Parisky K. M. Agosto J. Pulver S. R. Shang Y. Kuklin E. Hodge J. J. L. (2008). PDF cells are a GABA-responsive wake-promoting component of the Drosophila sleep circuit. Neuron 60, 672–682. 10.1016/j.neuron.2008.10.042 19038223 PMC2734413

[B56] Picot M. Cusumano P. Klarsfeld A. Ueda R. Rouyer F. (2007). Light activates output from evening neurons and inhibits output from morning neurons in the Drosophila circadian clock. PLoS Biol. 5, e315–e2521. 10.1371/journal.pbio.0050315 18044989 PMC2229858

[B57] Pitman J. L. McGill J. J. Keegan K. P. Allada R. (2006). A dynamic role for the mushroom bodies in promoting sleep in Drosophila. Nature 441, 753–756. 10.1038/nature04739 16760979

[B58] Pollack I. Hofbauer A. (1991). Histamine-like immunoreactivity in the visual system and brain of *Drosophila melanogaster* . Cell Tissue Res. 266, 391–398. 10.1007/BF00318195 1684918

[B59] Qian Y. Cao Y. Deng B. Yang G. Li J. Xu R. (2017). Sleep homeostasis regulated by 5HT2b receptor in a small subset of neurons in the dorsal fan-shaped body of drosophila. Elife 6, e26519. 10.7554/eLife.26519 28984573 PMC5648528

[B60] Raghu S. V. Borst A. (2011). Candidate glutamatergic neurons in the visual system of drosophila. PLoS One 6, 194722–e19511. 10.1371/journal.pone.0019472 PMC3088675 21573163

[B61] Redlin U. (2001). Neural basis and biological function of masking by light in mammals: Suppression of melatonin and locomotor activity. Chronobiol. Int. 18, 737–758. 10.1081/CBI-100107511 11763983

[B62] Robinson J. E. Paluch J. Dickman D. K. Joiner W. J. (2016). ADAR-mediated RNA editing suppresses sleep by acting as a brake on glutamatergic synaptic plasticity. Nat. Commun. 7, 10512. 10.1038/ncomms10512 26813350 PMC4737855

[B63] Schlichting M. Weidner P. Diaz M. Menegazzi P. Dalla Benetta E. Helfrich-Förster C. (2019). Light-Mediated circuit switching in the Drosophila neuronal clock network. Curr. Biol. 29, 3266–3276. 10.1016/j.cub.2019.08.033 31564496

[B64] Sehgal A. Price J. Young M. W. (1992). Ontogeny of a biological clock in *Drosophila melanogaster* . Proc. Natl. Acad. Sci. U. S. A. 89, 1423–1427. 10.1073/pnas.89.4.1423 1741397 PMC48463

[B65] Shang Y. Donelson N. C. Vecsey C. G. Guo F. Rosbash M. Griffith L. C. (2013). Short neuropeptide F is a sleep-promoting inhibitory modulator. Neuron 80, 171–183. 10.1016/j.neuron.2013.07.029 24094110 PMC3792499

[B66] Shang Y. Griffith L. C. Rosbash M. (2008). Light-arousal and circadian photoreception circuits intersect at the large PDF cells of the Drosophila brain. Proc. Natl. Acad. Sci. U. S. A. 105, 19587–19594. 10.1073/pnas.0809577105 19060186 PMC2596742

[B67] Shang Y. Haynes P. Pírez N. Harrington K. I. Guo F. Pollack J. (2011). Imaging analysis of clock neurons reveals light buffers the wake-promoting effect of dopamine. Nat. Neurosci. 14, 889–895. 10.1038/nn.2860 21685918 PMC3424274

[B68] Shaw P. J. Cirelli C. Greenspan R. J. Tononi G. (2000). Correlates of sleep and waking in *Drosophila melanogaster* . Science 287, 1834–1837. 10.1126/science.287.5459.1834 10710313

[B69] Sheeba V. Chandrashekaran M. K. Joshi A. Sharma V. K. (2002). Developmental plasticity of the locomotor activity rhythm of *Drosophila melanogaster* . J. Insect Physiol. 48, 25–32. 10.1016/S0022-1910(01)00139-1 12770129

[B70] Sheeba V. Fogle K. J. Kaneko M. Rashid S. Chou Y. T. Sharma V. K. (2008a). Large ventral lateral neurons modulate arousal and sleep in Drosophila. Curr. Biol. 18, 1537–1545. 10.1016/j.cub.2008.08.033 18771923 PMC2597195

[B71] Sheeba V. Gu H. Sharma V. K. O’Dowd D. K. Holmes T. C. (2008b). Circadian- and light-dependent regulation of resting membrane potential and spontaneous action potential firing of Drosophila circadian pacemaker neurons. J. Neurophysiol. 99, 976–988. 10.1152/jn.00930.2007 18077664 PMC2692874

[B72] Sinakevitch-Pean I. Geffard M. Plotnikova S. I. (2001). Localization of glutamate in the nervous system of the fly *Drosophila melanogaster*: An immunocytochemical study. J. Evol. Biochem. Physiol. 37, 83–88. 10.1023/A:1017574120553 11424530

[B73] Sitaraman D. Aso Y. Rubin G. M. Nitabach M. N. (2015). Control of sleep by dopaminergic inputs to the drosophila mushroom body. Front. Neural Circuits 9, 73. 10.3389/fncir.2015.00073 26617493 PMC4637407

[B74] Stoleru D. Peng Y. Agosto J. Rosbash M. (2004). Coupled oscillators control morning and evening locomotor behaviour of Drosophila. Nature 431, 862–868. 10.1038/nature02926 15483615

[B75] Szuperak M. Churgin M. A. Borja A. J. Raizen D. M. Fang-Yen C. Kayser M. S. (2018). A sleep state in Drosophila larvae required for neural stem cell proliferation. Elife 7, 332200–e33319. 10.7554/eLife.33220 PMC5834245 29424688

[B76] Takemura S. Y. Karuppudurai T. Ting C. Y. Lu Z. Lee C. H. Meinertzhagen I. A. (2011). Cholinergic circuits integrate neighboring visual signals in a drosophila motion detection pathway. Curr. Biol. 21, 2077–2084. 10.1016/j.cub.2011.10.053 22137471 PMC3265035

[B77] Tomita J. Ban G. Kato Y. S. Kume K. (2021). Protocerebral bridge neurons that regulate sleep in *Drosophila melanogaster* . Front. Neurosci. 15, 647117–647215. 10.3389/fnins.2021.647117 34720844 PMC8554056

[B78] Tomita J. Ueno T. Mitsuyoshi M. Kume S. Kume K. (2015). The NMDA Receptor promotes sleep in the fruit fly, drosophila melanogaster. PLoS One 10, e0128101. 10.1371/journal.pone.0128101 26023770 PMC4449117

[B79] Ueno T. Tomita J. Tanimoto H. Endo K. Ito K. Kume S. (2012). Identification of a dopamine pathway that regulates sleep and arousal in Drosophila. Nat. Neurosci. 15, 1516–1523. 10.1038/nn.3238 23064381

[B80] VanVickle-Chavez S. J. Van Gelder R. N. (2007). Action spectrum of Drosophila cryptochrome. J. Biol. Chem. 282, 10561–10566. 10.1074/jbc.M609314200 17284451

[B81] Vogt K. Aso Y. Hige T. Knapek S. Ichinose T. Friedrich A. B. (2016). Direct neural pathways convey distinct visual information to Drosophila mushroom bodies. eLife 5, 1–13. 10.7554/eLife.14009 PMC4884080 27083044

[B82] Yao Z. Shafer O. T. (2014). The Drosophila circadian clock is a variably coupled network of multiple peptidergic units. Science 343, 1516–1520. 10.1126/science.1251285 24675961 PMC4259399

[B83] Yasuyama K. Meinertzhagen I. A. (1999). Extraretinal photoreceptors at the compound eye’s posterior margin in *Drosophila melanogaster* . J. Comp. Neurol. 412, 193–202. 10.1002/(sici)1096-9861(19990920)412:2<193::aid-cne1>3.0.co;2-0 10441750

[B84] Yasuyama K. Salvaterra P. M. (1999). Localization of choline acetyltransferase-expressing neurons in Drosophila nervous system. Microsc. Res. Tech. 42, 65–79. 10.1002/(SICI)1097-0029(19990415)45:2<65::AID-JEMT2>3.0.CO;2-0 10332725

[B85] Yuan Q. Joiner W. J. Sehgal A. (2006). A sleep-promoting role for the Drosophila serotonin receptor 1A. Curr. Biol. 16, 1051–1062. 10.1016/j.cub.2006.04.032 16753559

[B86] Yuan Q. Lin F. Zheng X. Sehgal A. (2005). Serotonin modulates circadian entrainment in Drosophila. Neuron 47, 115–127. 10.1016/j.neuron.2005.05.027 15996552

[B87] Zhang L. Lear B. C. Seluzicki A. Allada R. (2009). The CRYPTOCHROME photoreceptor gates PDF neuropeptide signaling to set circadian network hierarchy in Drosophila. Curr. Biol. 19, 2050–2055. 10.1016/j.cub.2009.10.058 19913424 PMC2805779

[B88] Zhao J. Warman G. R. Stanewsky R. Cheeseman J. F. (2019). Development of the molecular circadian clock and its light sensitivity in Drosophila melanogaster. J. Biol. Rhythms 34, 272–282. 10.1177/0748730419836818 30879378

